# Extramammary Paget’s Disease of the Suprapubic Region in a Male: A Novel Diagnostic Imaging Approach and Literature Review

**DOI:** 10.3390/jcm15010160

**Published:** 2025-12-25

**Authors:** Piotr Sobolewski, Mateusz Koper, Malgorzata Kolos, Irena Walecka

**Affiliations:** 1 Dermatology Clinic, National Medical Institute of the Ministry of Interior and Administration, 02-507 Warsaw, Poland; 2 Chair and Clinic of Dermatology and Pediatric Dermatology, Centre of Postgraduate Medical Education, 02-507 Warsaw, Poland; 3 Pathomorphological Center, National Institute of Medicine of the Ministry of Interior and Administration, 02-507 Warsaw, Poland

**Keywords:** extramammary Paget’s disease, line-field confocal optical coherence tomography, videodermoscopy, non-invasive imaging

## Abstract

Extramammary Paget’s disease (EMPD) is a rare cutaneous adenocarcinoma typically arising on apocrine gland-rich skin. This suprapubic location is exceptionally rare. Its nonspecific erythematous plaques often mimic benign inflammatory or infectious dermatoses, delaying diagnosis. We report an 80-year-old male who presented with a chronic suprapubic plaque. Videodermoscopy and line-field confocal optical coherence tomography (LC-OCT) highlighted irregular vascular patterns and pagetoid cells, raising suspicion for EMPD and guiding a biopsy. Histopathology confirmed carcinoma in situ, and immunostains (CK7 positive, CK20 negative) supported a primary cutaneous origin. Comprehensive screening ruled out associated malignancies; however, guidelines note that colon, rectal, prostate, and bladder cancers are the most frequent synchronous tumors and suggest considering tailored internal malignancy screening. Wide local excision achieved clear margins; after one year, there is no recurrence. The literature indicates that recurrence remains frequent after surgery and may not correlate with margin width, necessitating careful long-term surveillance. For patients unfit for surgery, alternative therapies include radiotherapy, topical imiquimod, and photodynamic therapy, though photodynamic therapy appears palliative rather than curative. Non-invasive imaging modalities, such as LC-OCT, provide high-resolution “virtual histology,” enhancing early diagnosis and reducing the need for repeated biopsies. Early recognition, appropriate staging, and multidisciplinary management are crucial for improving outcomes.

## 1. Introduction

Extramammary Paget’s disease (EMPD) is a rare intraepithelial adenocarcinoma that primarily affects apocrine gland-bearing skin, most commonly the vulvar, perianal, and genital regions [1]. First described by Crocker in 1889 [2], EMPD represents a distinct clinical and pathological entity from mammary Paget’s disease, although both share histopathological features, including the presence of large, pale-staining Paget cells within the epidermis [3]. EMPD predominantly affects postmenopausal women, particularly in the vulvar region [4]. EMPD is a rare cutaneous adenocarcinoma that typically arises in older adults (around 50–80 years of age). Notably, its incidence and gender predilection vary by region and ethnicity: Western cohorts show a female predominance (EMPD is 2–7 times more common in women), whereas Asian populations show a male predominance (about 3.5:1 in favor of men). Indeed, Asian males have the highest reported incidence of EMPD. Commonly affected sites are the vulva in women and the penoscrotal or adjacent truncal areas in men; by contrast, cases in atypical locations (such as the suprapubic region) are seldom documented. This epidemiologic profile highlights why reporting a rare male case in an unusual location (suprapubic skin) is particularly valuable for the medical literature [5].

Clinically, EMPD often presents as a slowly enlarging, erythematous, pruritic, and eczematous lesion, frequently leading to delayed diagnosis due to its nonspecific appearance and resemblance to benign dermatologic conditions such as dermatitis or fungal infections [5]. Histopathological examination remains the gold standard for diagnosis [6], with immunohistochemical staining aiding in differentiating primary EMPD from secondary involvement by underlying adnexal or visceral malignancies [7]. In addition to a primary intraepidermal origin, EMPD-like lesions may also result from epidermotropic spread of malignant cells or direct extension from an underlying internal carcinoma—secondary EMPD [8].

While surgical excision remains the mainstay of treatment for localized disease, recurrence rates are high due to subclinical extension of tumor beyond visible margins [9,10]. In invasive cases, regional or distant metastases can occur and portend a poorer prognosis. Current evidence-based guidelines, therefore, recommend age- and site-appropriate screening for underlying malignancies at baseline to distinguish primary vs. secondary EMPD. Moreover, due to the chronic and recurrent nature of EMPD, investigation into novel diagnostic methods and therapies is ongoing to improve management of this challenging malignancy [11].

## 2. Case Presentation

An 80-year-old male with a history of chronic comorbidities presented with a persistent erythematous and pruritic lesion located in the suprapubic region. The lesion had been present for several months and had gradually enlarged despite treatment with topical corticosteroids and antifungal agents administered under the initial presumption of dermatitis. On clinical examination, a well-demarcated erythematous plaque with superficial erosion and scaling, measuring approximately 7 × 6 cm, was observed on the suprapubic skin above the pubic symphysis. No inguinal lymphadenopathy was detected.

Videodermoscopy revealed a sharply circumscribed erythematous area with irregular whitish scaling and superficial erosion, suggestive of an intraepidermal neoplastic process. Line-field confocal optical coherence tomography (LC-OCT) further demonstrated the presence of large, atypical cells with abundant pale cytoplasm distributed within the epidermis, corresponding to Paget cells, and confirmed the dermoscopic suspicion. A punch biopsy was subsequently performed, and histopathological analysis was conducted. The haematoxylin and eosin-stained section demonstrated marked histopathological changes within the epidermis. There was a prominent infiltration of epidermis by large, atypical cells, occurring both singly and in cohesive nests. The cells were distinguished by abundant, pale cytoplasm and large, pleomorphic nuclei. Some nuclei appeared vesicular with prominent nucleoli. The cells were scattered throughout all layers of epidermis in a “pagetoid” distribution. The underlying dermis exhibited superficial, perivascular inflammatory infiltrate. No invasion of dermal stroma was evident. Immunohistochemical staining for Cytokeratin 7 demonstrated strong and diffuse cytoplasmic positivity within the large, atypical intraepidermal cells.

**Figure 1:**
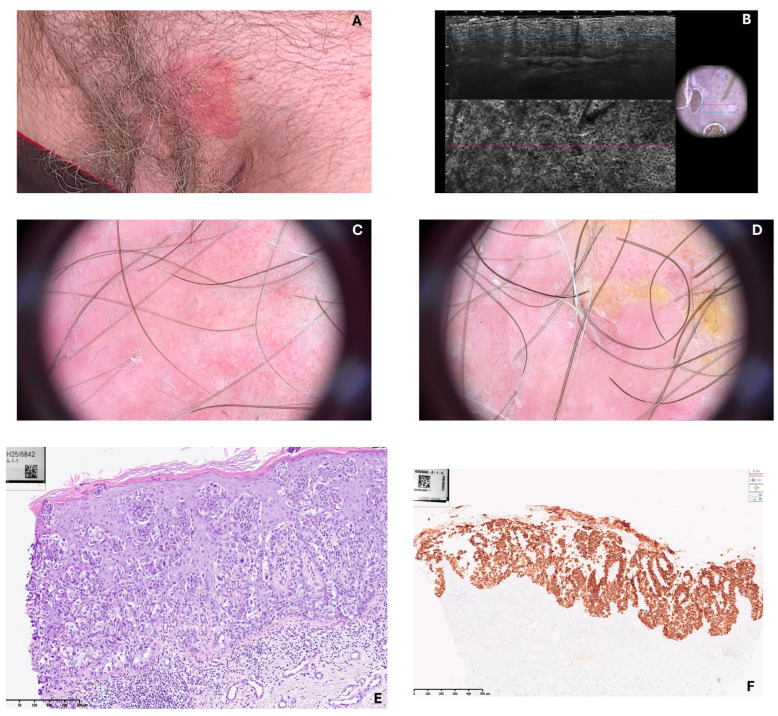
(**A**)—macroscopic image of the lesion—erythematous infiltrative plaque in suprapubic lesion; (**B**)—line-field confocal optical coherence tomography (LC-OCT) image—more details on Figure 2; (**C**,**D**)—videodermoscopic images of the lesion—a sharply circumscribed erythematous area with irregular whitish scaling and superficial erosion, numerous dotted vessels; (**E**)—histopathological image, hematoxylin and eosin staining (HE)—a prominent infiltration of epidermis by large, atypical cells, occurring both singly and in cohesive nests; (**F**)—histopathological image, positive cytoplasmic reaction for cytokeratin 7 (CK7+).

**Figure 2:**
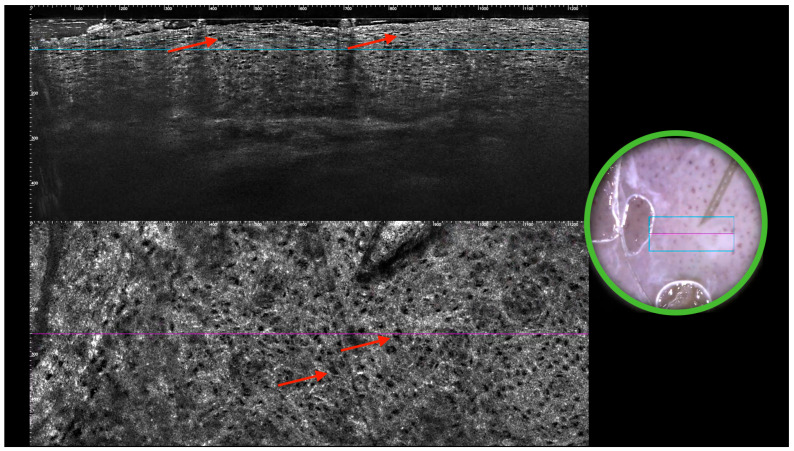
LC-OCT image—in green circle, dermoscopic in vivo view of examined lesion, purple and blue lines indicate the examined portion of the lesion within the dermoscopic colocalized site. The red arrows indicate big keratinocytes ascending into the epidermis—the pagetoid infiltration.

In sharp contrast, the adjacent non-neoplastic keratinocytes of the epidermis were negative. The staining pattern effectively highlighted the full extent of the neoplastic infiltration and confirmed the Paget cells, a finding characteristic of this disease. Representative videodermoscopy, LC-OCT, and histopathology images are provided (Figure 1 and Figure 2).

The patient subsequently underwent wide local excision with appropriate clinical margins until histopathologically free of lesions. The postoperative course was uneventful. The patient continues to be monitored under regular dermatological surveillance (videodermoscopy) for over 1 year now with no signs of recurrence.

The patient was also examined to exclude other malignancies: chest X-ray, abdomen and lymph node ultrasound as well as tumor blood markers tests (CA-125, CA 19-9, CEA, AFP, and PSA) were negative—supporting the classification of primary (cutaneous) EMPD.

## 3. Management, Treatment Options, and Follow-Up

Surgery: Wide local excision (WLE) of the visible lesion with clear histologic margins remains the standard first-line treatment for localized EMPD. Intraepidermal (in situ) disease is effectively managed with complete excision; however, achieving negative margins can be challenging due to microscopic peripheral spread. Conventional excisions using 1–2 cm clinical margins often have high recurrence rates, reported in some series to be in the range of 20–60% [12]. Mohs micrographic surgery (MMS) offers tissue-sparing serial excision with margin evaluation and has demonstrated lower relapse rates and longer relapse-free survival compared to WLE [9]. MMS is, therefore, considered by many experts to be the optimal surgical approach for EMPD when available, particularly for ill-defined or recurrent lesions. In our case, WLE achieved clear margins and has been successful to date, but the literature indicates that Mohs surgery can reduce the likelihood of residual disease and recurrence. For invasive EMPD that has penetrated into the dermis, surgical resection with clear margins is also the preferred treatment, potentially combined with lymph node evaluation. Complete lymph node dissection is generally performed if there is clinical or biopsy-proven nodal metastasis, though its impact on overall survival is unproven [8]. Notably, routine prophylactic sentinel lymph node biopsy in the absence of clinical lymphadenopathy is not recommended for EMPD, according to recent guidelines [12]. Instead, surgery is focused on the primary site, and nodes are addressed only if clinically indicated.

Radiation Therapy: Radiotherapy is an alternative treatment for patients who are not surgical candidates or who have unresectable disease. Multiple case reports and small series have shown that external beam radiation can achieve good local control of primary EMPD. In one retrospective study of 41 patients treated with radiation (median dose ~60 Gy), the 5-year local progression-free rate was ~82%, with minimal high-grade toxicity. Radiation may be especially useful for treating areas where surgery would be mutilating or for patients with medical comorbidities precluding surgery. It can also be considered as adjuvant therapy if margins are positive and re-excision is not feasible. The downside is that radiation, like surgery, may not prevent recurrence if there is a field of subclinical disease, and treating large areas can risk chronic radiation dermatitis. Nonetheless, the safety and efficacy profile of modern radiotherapy makes it a reasonable non-invasive option in select cases [8].

Topical Imiquimod: Imiquimod 5% cream, an immune response modifier, has been used off-label to treat superficial EMPD with some success. Studies have documented complete clinical response rates on the order of ~50–70% after prolonged courses of topical imiquimod, but with a significant proportion of responders later experiencing recurrence. For example, Sawada et al. reported an initial 56% complete response rate in EMPD treated with imiquimod, yet 60% of those complete responders had disease recurrence after a period of remission. Imiquimod is typically applied 3–5 times per week for several months; evidence suggests that longer durations (up to 6 months) improve response rates. This therapy can be useful for patients who cannot or will not undergo surgery, or as an adjunct to reduce tumor burden before surgery in extensive cases. However, close monitoring is required, as there are reports of EMPD progressing to invasive disease during or after imiquimod therapy in some patients. The need for long treatment courses and the risk of incomplete eradication mean imiquimod is generally reserved for in situ disease in carefully selected cases, with surgical excision still considered the definitive treatment when possible. Long-term follow-up is mandatory if topical therapy is used as monotherapy [13].

Photodynamic Therapy (PDT): Photodynamic therapy is a non-surgical treatment that involves application of a photosensitizing agent (such as 5-aminolevulinic acid) to the lesion, followed by illumination with a specific light wavelength to induce a cytotoxic reaction in the tumor cells. PDT has been tried in EMPD, with multiple case reports documenting partial or complete responses. The appeal of PDT is its non-invasive nature; however, overall outcomes suggest PDT is usually not curative in EMPD but may be useful for palliation or for patients who cannot undergo surgery. Some studies have reported good short-term results, but recurrences are common. PDT can reduce lesion size and alleviate symptoms (pruritus, irritation) and thus might be employed to improve quality of life or as an adjunct to other therapies. Its side effects include significant pain during illumination and post-treatment photosensitivity in the treated area. Given these limitations, PDT is considered a second-line or adjunct modality in the management of EMPD, not a front-line curative treatment [14].

Systemic and Targeted Therapies: For metastatic or unresectable EMPD, systemic therapy is indicated, although no consensus regimen exists due to the rarity of advanced cases. Traditional cytotoxic chemotherapies (e.g., cisplatin or carboplatin combined with 5-FU, or taxane-based regimens) have been employed, but responses are modest and often transient [15]. In recent years, attention has turned to targeted therapies and immunotherapies. Notably, a high proportion of primary EMPD lesions overexpress the HER2/neu (ERBB2) protein. Multiple case reports have shown that HER2-positive metastatic EMPD can respond dramatically to anti-HER2 monoclonal antibody therapy (trastuzumab), often in combination with chemotherapy. Ongoing clinical trials are evaluating agents like trastuzumab (with or without docetaxel) in advanced EMPD, and this targeted approach is emerging as a promising option for patients whose tumors demonstrate HER2 overexpression [16]. Immunotherapy with checkpoint inhibitors (e.g., anti-PD-1 or anti-PD-L1 agents) has been explored anecdotally in EMPD, especially in tumors with high microsatellite instability or high tumor mutational burden. However, unlike melanoma, most EMPD tumors do not strongly express PD-L1, and the efficacy of checkpoint blockade in EMPD has not been clearly established. Overall, management of metastatic EMPD is individualized, often borrowing from protocols used in analogously behaving adenocarcinomas. Given the generally poor prognosis of disseminated EMPD and lack of large trials, enrollment in clinical studies or compassionate use of targeted agents is encouraged when feasible [17].

Follow-Up: All patients with EMPD, regardless of treatment modality, require long-term follow-up due to the high risk of recurrence. EMPD is characterized by frequent local recurrences, even after initially successful treatment. Published recurrence rates vary, but even with optimal therapy, recurrences can occur in 20–50% of cases and may present many years after the initial treatment. Therefore, a prolonged surveillance period is warranted. Current expert guidelines recommend close follow-up (every 3–6 months) for at least the first 5 years after treatment, since most recurrences happen in this window [12]. Follow-up visits typically include careful clinical examination of the affected area and regional lymph nodes. Non-invasive tools like dermoscopy can be extremely useful during follow-up to evaluate any scar or remnant area for signs of recurrence (e.g., the reappearance of vascular patterns or milky-red coloration). As previously noted, emerging modalities like LC-OCT may enhance the ability to detect subclinical recurrence during follow-up [18]. Patients should also be counseled about the symptoms and signs of recurrence and the importance of returning for evaluation if they notice any new changes. In cases of secondary EMPD, follow-up must additionally coordinate with appropriate specialists (e.g., colorectal or urologic oncology) managing the internal malignancy. Ultimately, diligent long-term surveillance is imperative, as early detection of recurrence or metastasis significantly improves the chances of successful secondary intervention.

## 4. Conclusions

Our combined case report and literature review highlight several key clinical insights. First, early diagnostic intervention—aided by tools like dermoscopy and LC-OCT—can significantly impact patient outcomes by confirming EMPD before further progression. Second, a multidisciplinary strategy is essential: dermatologists, oncologists, pathologists, and surgeons should collaborate to ensure appropriate workup, including internal malignancy screening as per updated guidelines, and to tailor therapy to the patients’ needs. Finally, given the propensity for recurrence and potential metastasis in EMPD, clinicians must commit to long-term surveillance and patient education. By integrating novel diagnostic techniques with established management principles, we can improve early detection and optimize care for this rare but challenging disease.

## Data Availability

Dataset available on request from the authors.

## Abbreviations

The following abbreviations are used in this manuscript:

EMPD	Extramammary Paget’s disease
LC-OCT	Line-field confocal optical coherence tomography
CK7	Cytokeratin 7
CA-125	Cancer Antigen 125
CA 19-9	Cancer Antigen 19-9
CEA	Carcinoembryonic Antige
AFP	Alpha-fetoprotein
PSA	Prostate-specific antigen
